# Endoscopic Management of Gastro-Entero-Pancreatic Neuroendocrine Tumours: An Overview of Proposed Resection and Ablation Techniques

**DOI:** 10.3390/cancers16020352

**Published:** 2024-01-13

**Authors:** Rocio Chacchi-Cahuin, Edward J. Despott, Nikolaos Lazaridis, Alessandro Rimondi, Giuseppe Kito Fusai, Dalvinder Mandair, Andrea Anderloni, Valentina Sciola, Martyn Caplin, Christos Toumpanakis, Alberto Murino

**Affiliations:** 1 Royal Free Unit for Endoscopy, The Royal Free Hospital and University College London (UCL) Institute for Liver and Digestive Health, London NW3 2QG, UK; rochio.chacchi@nhs.net (R.C.-C.);; 2 Department of HPB Surgery and Liver Transplant, Royal Free Hospital NHS Foundation Trust, London NW3 2QG, UK; 3 Neuroendocrine Tumour Unit, The Royal Free Hospital, Pond Street, London NW3 2QG, UK; 4 Gastroenterology and Endoscopy Unit, Fondazione IRCCS Policlinico San Matteo, 27100 Pavia, Italy; a.anderloni@smatteo.pv.it; 5 Gastroenterology and Endoscopy Unit, Fondazione IRCCS Ca’ Granda Ospedale Maggiore Policlinico Milano, 20122 Milan, Italy; valentinasciola@libero.it

**Keywords:** NET, endoscopy, ESD, EMR, ablation

## Abstract

**Simple Summary:**

Neuroendocrine tumours (NETs) are relatively rare gastrointestinal neoplasms. Many NETs have a favourable prognosis, but some show aggressive features and poor long-term survival. A relatively higher incidence of small lesions amenable to endoscopic resection has been noted. The aim of this review is to present a thorough review of the literature to assist clinicians in the endoscopic management of neuroendocrine tumours (rectal, gastric, duodenal, pancreatic and oesophageal NETs), to highlight novel endoscopic therapeutic techniques of resection.

**Abstract:**

A literature search of MEDLINE/PUBMED was conducted with the aim to highlight current endoscopic management of localised gastro-entero-pancreatic NETs. Relevant articles were identified through a manual search, and reference lists were reviewed for additional articles. The results of the research have been displayed in a narrative fashion to illustrate the actual state-of-the-art of endoscopic techniques in the treatment of NETs. Localised NETs of the stomach, duodenum and rectum can benefit from advanced endoscopic resection techniques (e.g., modified endoscopic mucosal resection, endoscopic full thickness resection, endoscopic submucosal dissection) according to centre expertise. Radiofrequency thermal ablation can be proposed as an alternative to surgery in selected patients with localised pancreatic NETs.

## 1. Introduction

Neuroendocrine tumours (NETs) are neoplasms derived from the diffuse neuroendocrine cell system. NETs can occur in different organs, including the pancreas, the duodenum, the stomach and the rectum, and they show a general relatively indolent growth rate together with the peculiar capacity to secrete a discrete range of active peptides and biogenic amines [1]. Gastro-entero-pancreatic neuroendocrine tumours (GEP-NETs) are still considered rare entities, although their incidence has significantly increased over the last 40 years. The age-adjusted incidence of GEP-NETs has gradually increased up to 3.65-fold in the United States and 3.8- to 4.8-fold in the United Kingdom. The largest increase occurred in gastric and rectal NETs, while the smallest increase occurred for small bowel NETs [2,3,4,5,6]. This increase in incidence is particularly evident for localized, low-grade tumours. Many studies found that even with a marked overall increase in incidence, the number of patients with distant metastases remained stable over 15 years. These data strongly suggest that increased NET incidence may be associated with the enhanced identification of small and asymptomatic lesions [5,6,7].

Regarding staging and biopsies, both conventional imaging and advanced imaging techniques, such as computed tomography (CT) scans, magnetic resonance imaging (MRI), and somatostatin receptor-based imaging (specifically, positron emission tomography (PET)/CT with 68 Ga-DOTA-peptides), are employed to accurately assess the disease and detect any possible distant metastases. If there is any indication of bone metastases on traditional imaging, it is recommended to conduct magnetic resonance imaging (MRI) of the spine and a 68 Gallium-positron emission tomography (PET) scan [8,9,10,11]. Considering the increased identification of small treatable lesions, our aim was to focus on the endoscopic treatment of oesophageal neuroendocrine tumours (O-NETs) gastric neuroendocrine tumours (G-NETs), duodenal neuroendocrine tumours (D-NETs), rectal neuroendocrine tumours (R-NETs) and pancreatic neuroendocrine tumours (P-NETs) that can be treated with endoscopic treatment. The focus on endoscopic treatment lies in its decreased invasiveness in comparison to conventional surgical methods while simultaneously guaranteeing similar effectiveness, as substantiated by recent medical research described hereafter.

## 2. Materials and Methods

We performed a literature search of EMBASE and MEDLINE databases, using the following keywords: rectal, rectum, gastric, duodenal, duodenum, oesophagus, oesophageal, pancreas, pancreatic, carcinoid, NET, therapy, endoscopy, mucosal resection, and submucosal dissection, to answer the following question “What endoscopic treatments are available for neuroendocrine tumours?”. An author reviewed the literature and identified the most relevant articles on this topic. Controversies related to case selection were discussed with two other reviewers, experts in advanced endoscopic resection techniques (EJD and AM).

## 3. Results

### 3.1. Oesophageal NETs

Oesophageal NETs represent only 0.2% of GEP-NETs. They are usually diagnosed incidentally as discrete polypoid lesions or in association with adenocarcinoma in Barrett’s oesophagus [12,13,14]. Endoscopic and histological features of O-NETs are not specific. In addition, due to the scarcity of evidence, guidelines for the treatment of O-NETs are missing, and physicians are mostly guided by local expertise and patient preference. 

In a publication by Schizas et al. [13], endoscopic resection has been proposed in O-NETs measuring less than 10 mm in size with the absence of regional lymph node metastases. This was also supported by Yazici et al. [14], who proposed a threshold of 10 mm as the maximum size recommended for the endoscopic resection of O-NETs. However, this indication is not supported by a large body of evidence but rather by the extrapolation of data from gastric and rectal NETs, which have shown higher rates of lymph node metastases for lesions measuring over 10 mm in size.

Although EMR allows for the en-bloc excision of small O-NETs, ESD appeared to be more accurate, allowing for a more accurate pathological examination of the respected specimen, which may not be obtained by EMR because of mucosal damage occurring during the resection [14] (Table 1).

### 3.2. Gastric NETs

**Figure 1:**
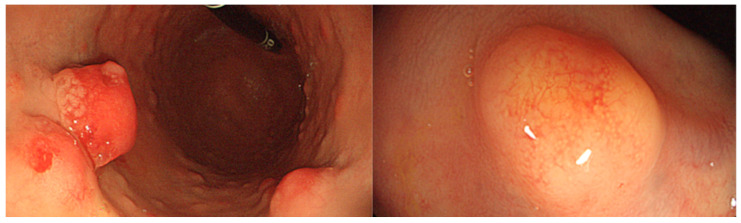
Example of type 1 gastric NET in the context of autoimmune atrophic gastritis on the left; example of rectal NET on the right.

G-NETs are classified into three categories according to the background gastric pathology: type 1 (prevalence 75%), type 2 (prevalence 5–10%), and type 3 (prevalence 15–25%) (Figure 1). Type 1 G-NETs are typically small, multiple, and associated with chronic atrophic gastritis with hypergastrinaemia and enterochromaffin-like cell hyperplasia. Type 2 G-NETs share the same pathological pathway due to excessive production of gastrin, although they are produced by a gastrinoma in the context of Zollinger–Ellison syndrome, usually in the setting of a multiple endocrine neoplasia type 1 (MEN-1). Type 3 G-NETs are sporadic lesions, typically solitary and undifferentiated; in addition, they are often larger in size when compared to type 1 and 2 G-NETs, and they occur in the setting of normal gastrin levels [9,10]. The tumour cell proliferation index allows for a further grading of G-NETs from G1 to G3 according to the World Health Organization (WHO) [15]. G1 has a mitotic count of <2 per 10 high-power fields (HPF) and/or Ki-67 ≤ 2%; G2 has a mitotic count of 2–20 per 10 HPF and/or Ki-67 3–20%; and G3 has a mitotic count and a Ki-67 > 20 [15,16].

#### 3.2.1. Type 1 Gastric NETs

Type 1 G-NETs occur more frequently in females because of their increased incidence of autoimmune chronic atrophic gastritis. Type 1 lesions are usually G1 tumours; thus, their metastatic risk is extremely low, and the prognosis is excellent. They are generally asymptomatic and usually incidentally detected during screening upper GI endoscopy [9,16,17,18,19,20]. The recent 2023 ENETS guidance paper listed ESD, EMR (standard and modified-EMR—m-EMR, with utilization of cap aspiration or with a ligation device or grasping forceps) and EFTR as possible treatments for localised, low-grade (G1 and G2), type 1 G-NETs [10]. Nevertheless, there are still no significant data documenting the superiority of any method [21]. Prospective studies comparing endoscopic resection methods are scarce, and the majority of data are provided mainly by retrospective studies [17].

A study with 62 patients and 87 type 1 G-NETs sized ≤10 mm, treated with ESD or EMR, compared their efficacy. The complete resection rate was higher when ESD was performed (94.9% vs. 83.3%, *p*-value = 0.174), although this was not statistically significant. However, a statistically significant difference was noted in the vertical margin involvement rate, which was lower in the ESD group (2.6% vs. 16.7%, *p* = 0.038). No difference was noted in the complication rate between the two groups [22].

A smaller study that included 13 type 1 G-NETs compared ESD and EMR as endoscopic resection techniques and measured effectiveness according to complete resection [23]. Seven ESDs and six EMRs were performed. The horizontal margins of excision were negative for all lesions, but the vertical margins were positive in four lesions (66.7%), all of them in the EMR group.

Recently, a large Korean study evaluated 103 patients with 114 tumours managed with EMR and ESD. En-bloc resection rates were similar, but complete resection was significantly higher in the ESD group. In addition, adverse event rates were similar among the two groups due to the need for surgical management and a disease-free survival rate [23].

A recent systematic review regarding the optimal endoscopic resection technique analysed 6 studies with 112 gastric type 1 NETs removed by EMR and 77 by ESD. Both methods appeared to have similar complete en-bloc resections, complications, and adverse event rates [21].

Surgical treatment is recommended only for patients with type 1 tumours that are predicted as T2 or lesions post-resection with positive margins; local wedge excision or partial gastrectomy should be considered (Table 2) [9,10].

It is noteworthy that, in patients with small (<10 mm) type 1 G-NETs who remained under endoscopic surveillance without resection, studies have shown no tumour-related deaths, even after a significant follow-up period (54–68 months). This conservative management seems to be a rational approach in selected patients with type 1 G-NETs, such as elderly patients with small tumours [24,25].

#### 3.2.2. Type 2 Gastric NETs

Type 2 G-NETs are linked with multiple endocrine neoplasia type 1 (MEN1) and Zollinger–Ellison syndrome (ZES). Approximately 13–37% of all patients with MEN1-ZES have been diagnosed with type 2 G-NET, while type 2 NETs are detected in only 0–2% of patients with sporadic ZES (without MEN1). Type 2 G-NETs are equally found in both male and female patients; around 30% of these lesions are metastatic at presentation. Additionally, patients with type 2 G-NETs have a lower survival rate than patients with type 1. Furthermore, although the majority of type 2 lesions are asymptomatic, the most common presenting symptom is related to peptic ulcers. This is due to the increased gastric acid secretion that can subsequently cause the development of peptic ulcers [26,27,28].

According to the ENETS guidelines, for type 2 G-NETs, treatment is usually dictated by the presence or not of additional duodenal and/or pancreatic lesions as part of MEN-1. Local or selective excision may be recommended, but this should be decided in multidisciplinary NETs centres of excellence [10]. The NCCN and the French Intergroup guidelines state that endoscopic resection may be indicated for lesions measuring up to 2 cm, and multiple biopsies of the surrounding mucosa are recommended for hypergastrinaemic patients [8,9]. However, because of their rarity, data on type 2 G-NETs are scarce; therefore, MDT discussion should always be encouraged.

#### 3.2.3. Type 3 Gastric NETs

Type 3 G-NETs are usually high-grade lesions (G3) with a tendency to infiltrate the muscularis propria, with a higher rate of lymphovascular involvement; this type of G-NETs is usually metastatic at presentation, with spreading to regional lymph nodes or liver. It often appears as a single lesion, usually greater than 10 mm. An atypical presentation (not serotonin related) of “carcinoid syndrome,” including flushing, tachycardia, and diarrhoea, occurs rarely in patients with gastric NETs (<1%) and is almost exclusively associated with type 3 tumours with liver metastasis [29,30,31].

ENETS guidelines recommend that type 3 G-NETs should be managed in the same way as gastric adenocarcinomas [10]. NCCN guidelines recommend radical gastric resection with perigastric lymph node dissection for localized type 3 G-NETs [8]. Interestingly, recent evidence has shown that carefully evaluated patients with small-size (<10 mm) and low-grade type 3 G-NETs could benefit from endoscopic resection [29,32,33]. A multicentre study gathering evidence from six tertiary referral centres found that endoscopic resection or limited surgical resection is feasible and safe in type 3 G-NETs under 10 mm that demonstrate a favourable grade (G1 or low G2) [34]. Nevertheless, MDT discussion in a tertiary referral centre is recommended before considering the endoscopic management of small and G1 type 3 G-NET [35].

### 3.3. Duodenal NETs

Duodenal NETs are rare, representing 2–4% of gastrointestinal NETs; they present as solitary lesions, confined to the duodenal submucosa [11], measuring different sizes. Endoscopic resection is recommended for D-NETs under 10 mm, although rare cases of local and distant metastases have been reported even for such small lesions [36]. In fact, D-NETs < 10 mm have a 14% rate of nodal metastasis, which increased to 47% for D-NETs measuring 21–50 mm in size. The invasion of the muscularis propria usually involves a size greater than 2 cm, and the presence of mitotic figures is an independent risk factor for metastasis [8,9,10].

D-NETs smaller than ≤10 mm confined to the submucosal layer and without lymph nodes or distant metastasis [8,9,10] are treated endoscopically when resection is required. Endoscopic resection in this setting is considered safe and effective. Conversely, surgical treatment is recommended for large (>20 mm) and/or metastatic D-NETs (Table 3).

Therapy is a subject of debate for non-functional, localised, well-differentiated (G1) D-NETs. Both endoscopic therapy and surgical interventions are seen as viable options in this particular scenario. However, there is a dearth of controlled studies evaluating the various techniques. Conversely, surgical intervention is advised for D-NETs that are bigger than 20 mm [8,9,10,27].

There are only a few retrospective studies that have addressed the endoscopic resection of D-NETs. One compared the outcomes of ligation-assisted endoscopic resection (EMR-L) and conventional EMR of 15 D-NETs with a mean tumour size of 6.6 ± 3.9 mm and mean procedure time of 11.0 ± 11.2 min. En-bloc resection and complete resection rates were higher in the ligation group (100% vs. 87.5%, and 85.7% vs. 62.5%, respectively), although this was not statistically significant. There was no evidence of local or distant metastasis at follow-up (26.1 ± 20.7 months) [37].

In another study by Fujimoto et al. [38], which included 10 patients with D-NETs treated with EMR-L, the en-bloc resection rate and endoscopic complete resection rates were 100%. Nevertheless, complete histopathological resection with clear margins was observed in only 70% of the specimens and vertical margins were negative in all 10. Three patients required additional surgical treatment because of lymphatic vessel invasion. No recurrence was identified at follow-up (mean period: 18.6 months). One major adverse event (perforation) was reported in one patient and was treated conservatively.

A recent retrospective study [36] evaluated the short- and long-term outcomes of ESD for non-ampullary D-NETs. Eight patients with G1 D-NETs with a diameter of less than 10 mm, restricted to the submucosal layer, and no lymph node involvement or distant metastases were included in the study. The majority of these lesions were in the duodenal bulb, with a median size of 6.4 mm. En-bloc resection, R0 resection, and curative resection had respectively 100%, 88%, and 88% success rates. Perforation occurred in one patient and was treated conservatively. Non-recurrencies were reported after a median follow-up period of 34 months.

In a multicentre retrospective study from 2019 [39], 60 non-ampullary D-NETs that underwent either endoscopic mucosal resection with a dual channel endoscope (EMR-D), EMR-L, EMR with a transparent cap (EMR-C), EMR with circumferential mucosal pre-cutting (EMR-P), or ESD were analysed and compared with surgical resection. For the group that received endoscopic treatment (EMR-D, EMR-L, EMR-C, EMR-P and ESD), en bloc resection, endoscopic full resection, and R0 rates were 88%, 92% and 50%, respectively. When the lesion size was more than 11 mm, the R0 rate was lower (50%, *p* = 0.003), and lymphovascular invasion was more common (33.3%, *p* = 0.043). The complete endoscopic resection rate was even lower (50%) in the NETs group with lesions ≥11 mm. After the histopathological analysis, patients who were treated surgically had a higher (90.9%) full resection rate than those who were treated endoscopically (50%), showing the advantage of surgical management for lesions larger than 10 mm.

Another new endoscopic technique is the band and slough technique (BAS), described by Hawa et al. [40], which is a minimally invasive endoscopic procedure for the management of small gastric and duodenal NETs (G-NETs and D-NETs). This is a variation of the band ligation procedure without the resection of the lesion. The BAS technique was used to treat three duodenal NETs and one type 1 G-NET, all of which were 10 mm in diameter. After the initial session of banding, both patients reached full recovery with no recurrence at the 3-month follow-up. Furthermore, 12-month monitoring of the site with biopsies revealed no tumour recurrence. The technique is relatively easy to perform; however, it does not provide a histological specimen and therefore its clear advantage in tumour eradication when compared with traditional resection techniques (i.e., m-EMR, ESD) is yet to be proven, and further numbers are therefore required [40].

Bourke et al. [41] conducted a retrospective study to assess patients with D-NETs who underwent ESD at three tertiary referral centres in Australia, France, and Belgium between 2012 and 2022. The results of the study indicated that en-bloc resection rates for D-NETs can reach 100% when performed by experienced endoscopists. There were no instances of distant metastatic spread or local recurrence in this study, suggesting that ESD could be a feasible alternative for patients with D-NETs measuring 10–15 mm who are not suitable candidates for surgery. To the best of our knowledge, only a single case series of three patients diagnosed with D-NETs and treated with EFTR can be found in the literature. The decision of EFTR was based on poor fitness for surgery. To note, disease-free survival at 1-year follow-up was documented [42].

Laparoscopic endoscopic cooperative surgery is an alternative way to achieve the full-thickness resection of D-NETs. The principle of this technique lies in associating the advantages of endoscopic resection (the ability to clearly demarcate the lesion and to avoid unnecessary bowel wall resection) with the suturing effectiveness of surgery. This technique has been adopted mainly in Eastern countries on single cases, with one case series reporting a curative resection in 85% of the cases [43].

### 3.4. Rectal NETs

The neuroendocrine tumours of the rectum represent 34% of all diagnosed GEP-NETs, and the rectum is the second site of localization by frequency after midgut (Figure 1). The incidence of R-NETs based on the Surveillance, Epidemiology, and End Results United States database (SEER) is approximately 1 per 100,000 population per year, accounting for 17.7% of all NETs [44,45,46].

As previously mentioned, a significant increase in the incidence in the R-NETs population has been observed; however, this was not followed by a higher incidence of distal metastasis, which remained stable. This scenario is likely to be explained by an improved and earlier diagnosis [7,44,45,46,47,48,49,50,51].

Grade is important when it comes to appointing a prognosis to a patient. Lower grades (G1–G2) are associated with better outcomes in terms of overall survival and risk of metastasis, often taking advantage of localised and endoscopic treatments [52,53]. In contrast, G3 grade is associated with the need for surgery, chemotherapy, and overall poor survival rates (10% at 5 years post-diagnosis) [54].

A systematic review by Mc Dermott et al. included 14 studies with 4575 patients; this showed that 80% of the R-NETs were <10 mm in size, 15% were between 10 and 20 mm, and 5% were >20 mm. Regional lymph nodal metastases were present in 8% of cases, and 4% of all patients had distant metastases. Tumour size greater than 10 mm and muscular and lymphovascular invasion were independently associated with increased risk of metastases. The 5-year survival rate was 93% in patients presenting with localised disease [50].

In their systematic review and meta-analysis, Xin Zhou et al. examined the differences between ESD, EMR, and modified ERM (m-EMR, an EMR performed with additional assistant devices like a ligation band or suction cap). The investigation spanned 650 patients and 10 retrospective studies. The results indicated that the ESD group had a higher rate of complete resection than the EMR group (RR 0.89 95% CI [0.79, 0.99]). In contrast, the rates of complete resection in both the ESD and m-EMR groups were similar (RR 1.03, 95% CI [0.95, 1.11]). Although the procedure duration in the ESD group was considerably longer than in the EMR group, the difference was not statistically significant (STD.50, 95% CI [−3.14, 0.14]). In the EMR group, local recurrence was observed in five cases, whereas no ESD patient experienced it [51].

These results were confirmed in a more recent meta-analysis by Zhang et al., which evaluated the treatment outcomes after ESD, m-EMR, and EMR for R-NETs < 16 mm. Compared with EMR, ESD achieved higher complete resection rates without increasing the overall complication rate (OR = 4.38, 95%CI: 2.43–7.91). Nevertheless, ESD was more time-consuming than EMR and m-EMR (respectively, MD = 6.72, 95%CI: 5.84–7.60 and MD = 12.21, 95%CI: 7.78–16.64). The m-EMR shared comparable outcomes with ESD for R-NETs < 16 mm. Both ESD and m-EMR were superior to conventional EMR in terms of complete resection rate without increasing safety concerns [52].

More recently, multiple retrospective studies comparing different endoscopic resection techniques for R-NETs < 10 mm, in the absence of deep invasion or lymphadenopathy, have been published [53,54,55,56]. A study evaluated 77 small rectal R-NET (≤10 mm) patients treated by m-EMR with endoscopic submucosal resection with band ligation (ESMR-L) (*n* = 53) or ESD (*n* = 24). En-bloc resection was achieved in all patients. A significantly higher histopathological complete resection rate was observed in the ESMR-L group (100%) than in the ESD group (54.2%) (*p* = 0.001). The procedure time of ESD was significantly longer than that of ESMR-L [55]. Another similar retrospective study from Japan, including 96 patients treated with EMR (*n* = 60), m-EMR (*n* = 21) and ESD (*n* = 21), showed similar results between the various resection techniques [57].

When endoscopy was compared with surgery in a large monocentric retrospective propensity-matched study of 104 patients, who were equally distributed between ESD and transanal endoscopic microsurgery (TEM) for R-NETs under 20 mm, a similar R0 resection rate was observed in the subgroup analysis divided by tumour size (<10 mm and 10 to 20 mm). However, a shorter procedure time and hospital stay for ESD patients (ESD 22 [range, 11–65] vs. TEM 35 [range, 17–160] minutes; ESD 2.5 [range, 1–5] vs. TEM 4 [range, 3–8] days) was noted [58].

Another technique that can be considered for the removal of R-NETs under 10 mm is endoscopic full-thickness resection. A multicentre retrospective study of 31 German centres reported 501 endoscopic procedures, of which 40 cases were R-NETs, using a full-thickness resection device (FTRD). The median lesion size was 8 mm and resection was endoscopically and histologically complete in all cases whereas full-thickness resection was achieved in 95% of cases. There were no major adverse events, and after follow-up endoscopy, no evidence of residual or recurrent tumour was reported [59].

In another recent study from Korea, 115 patients with R-NETs (<10 mm) were included. Rectal NETs were either removed by ESD (*n* = 79) or underwater endoscopic mucosal resection (UEMR) (*n* = 36). There was no difference in terms of R0 resection rate between the UEMR and ESD groups (86.1% vs. 86.1%, *p* = 0.996), whereas the procedure time was significantly shorter with UEMR (5.8 ± 2.9 vs. 26.6 ± 13.4 min, *p* < 0.001) [55].

According to clinical management guidelines, surgical resection with the removal of associated lymphatic tissue is the preferred treatment for R-NETs greater than 20 mm because of the high risk of lymphatic invasion and metastasis [9,46]. The existence of predictors of nodal involvement, including a tumour size ≥ 15 mm, the atypical endoscopic aspect, muscular layer invasion, a tumour grade of G2-G3, and lymphovascular invasion should guide the management of R-NETs. A low anterior rectal resection with complete mesorectal excision should be performed when one or more of these characteristics are present [9,60,61].

To summarize, in R-NETs < 15 mm, advanced resection techniques (i.e., m-EMR, ESD, EFTR) must be preferred to standard polypectomy or simple endoscopic mucosal resection (EMR), which should be avoided because of the low rate of complete resection. In particular, ESD could be considered as a first-line treatment for fibrotic lesions and should be selected over m-EMR for lesions over 10 mm in diameter as it appears to achieve better complete resection rate outcomes (Table 4) [9,60,61].

### 3.5. Pancreatic NETs

Pancreatic neuroendocrine tumour (P- NET) prevalence is 10% of all pancreatic neoplasms. Their prognosis is good, even in an advanced disease setting [9,62,63,64].

P-NETs are classified as either sporadic or genetically determined when they occur in the context of inherited syndromes. Additionally, they are categorized according to the manifestation or non-appearance of symptoms caused by hormone secretion, such as insulin, gastrin, and glucagon, which are secreted by functional P-NETs [61,62]. Functional pancreatic nanotubes (P-NETs) are typically detected during the early stages, when the lesions are still minor [42]. In the majority of cases, surgery is the preferred treatment option for these tumours. Conversely, the detection of non-functional P-NETs typically occurs at a later stage; however, the widespread application of CT and MRI imaging has substantially augmented the detection rate of minor incidental lesions [63,64,65].

Surgery is the most common treatment for P-NETs, but it is associated with potential perioperative risks. Specifically, postoperative complications can be more frequent than those after surgery for ductal adenocarcinoma [66]. For non-functioning P-NETs, the therapeutic approach depends on tumour localizations and size. The ENETs guidelines recommend observation and surveillance for well-differentiated grade 1 tumours <2 cm [61]. However, the interim analysis of the ASPEN trial (a prospective international multicentre study longitudinally following patients with P-NETs <2 cm, either monitored or resected) suggested a more personalized management for non-functional P-NETs between 1 and 2 cm and a mandatory surgical resection for all lesions with a dilated main pancreatic duct, as most of them exhibit a more aggressive biological behaviour [67].

Parenchyma-preserving surgery is preferable for small lesions, mainly those in the head of the pancreas, with a limiting factor being the proximity of the tumour to the pancreatic duct. Alternatively, pancreatoduodenectomy with or without pylorus preservation (Whipples vs. PPPD) and distal pancreatectomy are the standard surgical procedures for P-NETs localized in the head and body/tail of the pancreas, respectively [64].

As an alternative, endoscopic ultrasound-guided radiofrequency ablation (EUS-RFA) has recently been described for functional smaller P-NETs [68,69]. Radiofrequency ablation (RFA) causes irreversible cellular damage, cellular apoptosis, and tissue coagulative necrosis by conveying high temperatures within the tumour mass [70,71]. Compared with surgical methods, the technical advantages of locoregional thermoablative techniques include the preservation of healthy surrounding tissues, lower rates of morbidity, shorter hospital stays, and reduced overall expenses. Furthermore, research suggests that immunomodulation may have a secondary anticancer effect [72].

Two devices are available for performing pancreatic RFA: a cooled needle connected to a dedicated energy source and a 1Fr probe that can be inserted into a 19G needle while being attached to a conventional energy source. Utilizing a high-frequency alternating current and EUS guidance, the needle is introduced into the designated lesion while maintaining a minimum distance of 2 mm from the pancreatic and biliary ducts to prevent injury or duct strictures. Doppler evaluation is employed to ensure that no harm is done to the vasculature [73].

In their recent study, Imperatore et al. [68] undertook a comprehensive review of the literature in order to determine whether EUS-guided RFA treatment is feasible, effective, and safe, and to identify P-NET characteristics that could serve as predictors of response to EUS-RFA. Sixty-nine patients were identified by the authors from twelve investigations. Males comprised thirty (49.2%) of the sixty-one patients, who were aged 65.4 years on average. The investigators identified 73 P-NETs, with a range of 4.5 to 40.0 mm in length, with the following locations: head (35.3%), body (39.7%), uncinate (8.8%), and tail (16.2%). The average measure of each was 16 mm. Out of the total, 30.1% (21 insulinomas and 1 VIPoma) were functional. P-NETs were administered an average of 1.3 RFA sessions over a follow-up period of 11 months (range: 1–34 months), with an overall effectiveness of 96% (75–100%). The response rate was found to be influenced by the size of the tumour. Specifically, larger tumours exhibited a higher frequency of failing to respond to treatment (mean size of 21.8 mm ± 4.71 in the non-response group vs. 15.07 mm ± 7.34 in the response group, *p* = 0.048). A P-NET size of less than 18 mm at EUS was associated with a positive response to EUS-RFA, as determined by the ROC curve, which had the following values: sensitivity (80%), specificity (78.6%), PPV (97.1%), and NPV (30.8%) [68].

A large series comparing EUS-RFA and surgery with propensity-score matching (1:1) in 89 patients affected by insulinoma has been recently published. Notably, clinical efficacy was comparable between RFA and surgery (95.5% vs. 100%, *p* = 0.16), but RFA outperformed surgery in terms of adverse event rate (18.0% vs. 61.8%, *p* < 0.001), severe adverse event rate (0.0% vs. 15.7%) and hospital stay (3.0 ± 2.5 vs. 11.1 ± 9.7, *p* < 0.001). However, recurrence was observed in EUS-RFA-treated patients [74].

A method based on EUS-guided ethanol injection has been described as an alternative to RFA for p-NET under 20 mm and G1-G2 grade without metastasis. A recent pilot study with five patients from Matsumoto et al. showed how a successful ablation could be achieved in four cases, with no recurrence and no adverse events reported [75]. A previous experience on 11 patients and 14 tumours by Park et al. reported a lower response rate (61.5%) with 3 cases of mild pancreatitis (30%) [76] (Table 5).

EUS-guided P–NET localization is another task that could be accomplished by gastrointestinal endoscopy. The localization consists of focal needle tattooing (EUS-FNT) or placing fiducial markers close to or inside lesions to make them easier to identify and resect during surgery. EUS-FNT was performed on a case series of 13 patients, 6 of whom had P-NETs, by labelling the pancreatic parenchyma within 3–5 mm of the lesion with sterile carbon-based ink to permit laparoscopic distal pancreatectomy (LDP). The surgeon’s ability to clearly view and palpate the lesion is restricted, if not non-existent, in LDP. Despite a mean of 20.3 days (range, 3–69) between EUS-FNT and surgery, all tattooed tissues were clearly visible at surgery [77].

Finally, when it comes to the diagnosis of P-NETs, fine-needle biopsy (FNB) is the technique recommended to obtain a specimen from pancreatic masses, although a meta-analysis encompassing 11 RCT failed to prove the statistical superiority of FNB over FNA (fine-needle aspiration) [78]. However, a recent network meta-analysis, including 16 studies for a total of 1934 patients, demonstrated how Franseen and Fork-tip needles (FNB), specifically those of the 22-gauge size, were the best performers in terms of obtaining tissue samples from pancreatic masses. At the same time, the level of confidence in the estimates was poor due to relatively few head-to-head trials supporting the comparisons and differences while carrying out the procedure that may have changed the final results [79].

## 4. Conclusions

Gastrointestinal neuroendocrine tumours are clinically diverse and rare neoplasms that can pose a treatment challenge for endoscopists. The most prevalent detection method for luminal NETs is endoscopy, and histopathology is the gold standard in their diagnosis. EUS has become an essential procedure for the staging and management of pancreatic NETs.

The best treatment depends on the location of the NETs, also requiring an accurate assessment of their size, local lymphadenopathy, and depth of invasion. Endoscopic resection techniques are evolving, with endoscopic submucosal dissection appearing to be an effective and safe method allowing for the en-bloc removal of lesions up to 2 cm in size, possessing an acceptable complete resection and a recurrence rate similar to surgery. The evaluation of the best technique to apply to an NET should be discussed in a multidisciplinary team meeting setting, and the discussion should consider tumour size and location as well as the patient’s preferences and local expertise.

Future evidence from a prospective randomised controlled trial is needed to better define the role of therapeutic endoscopy in the resection of luminal NETs measuring up to 2 cm and to investigate whether this could eventually replace surgical intervention when the risk of lymphovascular invasion is low.

## Figures and Tables

**Table 1:** Endoscopic management of well-differentiated oesophageal neuroendocrine tumours.

Size	Proposed Treatments	Body of Evidence	Pros and Cons
Oesophageal NET < 10 mm	ESD	Case report	Little evidence to support the data.

**Table 2:** Endoscopic management of gastric neuroendocrine tumours.

Size	Proposed Treatments	Body of Evidence	Pros and Cons
Gastric NET Type 1 < 10 mm	ESD m-EMR (cap- or ligation-assisted) Follow up	Multicentre retrospective study Retrospective studies	Slight advantage of ESD over m-EMR in complete resection rates and free vertical margins. No differences in terms of rates of complication. Some evidence for conservative treatment “watch and wait” in G1 NETs.
Gastric NET Type 2 < 10 mm	ESD m-EMR (cap- or ligation-assisted)	Consensus	Same as type 1 gastric NETs.
Gastric NET Type 3	Surgery	Consensus	

**Table 3:** Endoscopic management of well-differentiated duodenal neuroendocrine tumours.

Size	Proposed Treatments	Body of Evidence	Pros and Cons
Duodenal NET < 10 mm	m-EMR EMR ESD Band-and-Slough Laparoscopic endoscopic cooperative surgery	Multicentre retrospective study Small-sample-size retrospective studies Case series	Acceptable rates of en-bloc resection and endoscopic full resection for m-EMR. Role of ESD is still to be decided (little supporting evidence). 50% of R0 in the larger cohorts of patients (90% in surgery patients). Only initial data for laparoscopic endoscopic cooperative surgery.

**Table 4:** Endoscopic management of well-differentiated rectal neuroendocrine tumours.

Size	Proposed Treatments	Body of Evidence	Pros and Cons
Rectal NET < 20 mm	ESD m-EMR (ligation-assisted) EMR FTRD	Systematic review and meta-analyses Retrospective multicentre studies	ESD and m-EMR are similar in terms of complete resection rates and complications for NETs under 10 mm. ESD has a longer procedure time compared to m-EMR. m-EMR with ligation devices showed a little benefit over ESD on vertical margin positivity. ESD and m-EMR are both superior to standard EMR in terms of complete resection. FTRD is feasible for R-NETs under 10 mm with a high complete resection rate. For NETs with a size from 10 mm to 20 mm, ESD should be proposed, with a careful evaluation of benefits over surgery techniques.

**Table 5:** Endoscopic management of well-differentiated pancreatic neuroendocrine tumours.

Size	Proposed Treatments	Body of Evidence	Pros and Cons
Pancreatic NET < 20 mm	Surveillance EUS-RFA EUS-alcohol injection Parenchyma-preserving surgery	Multicentre retrospective study Systematic review with meta-analysis Case reports	Surveillance can be proposed for patients not fit for surgery with non-functioning small P-NETs (<10 mm) EUS-RFA can be considered for P-NETs < 20 mm Parenchyma-preserving surgery is considered the standard of care for fit-for-surgery patients with P-NETs Paucity of data to support EUS-alcohol injection
